# A Data Report on the Curation and Development of a Database of Genes for Acute Respiratory Distress Syndrome

**DOI:** 10.3389/fgene.2021.750568

**Published:** 2021-12-09

**Authors:** Erick Quintanilla, Kimberly Diwa, Ashley Nguyen, Lavang Vu, Inimary T. Toby

**Affiliations:** University of Dallas, Department of Biology, Irving, TX, United States

**Keywords:** acute respiratory distress (ARDS), genes, variants, database (DB), chromosome location, biological insights and machine learning

## Introduction

Acute respiratory distress syndrome (ARDS) is a syndrome of hypoxic respiratory failure characterized by diffuse pulmonary infiltrates and accumulation of protein-rich pulmonary edema that cause reduction in lung compliance alveolar collapse and ventilation-perfusion mismatch (Katzenstein et al., 1976; Ware and Matthay, 2000; Rubenfeld et al., 2005; Matute-Bello et al., 2008; Phua et al., 2009; Force et al., 2012). ARDS affects approximately 190,600 patients per year in the United States, with mortality up to 45% (Wellman et al., 2016). Despite improvements in intensive care during the last fifteen years, ARDS is still the major cause of mortality and morbidity in intensive care (Katzenstein et al., 1976; Ware and Matthay, 2000; Matute-Bello et al., 2008; Force et al., 2012; Wellman et al., 2016). In fact, ARDS therapy has seen limited progress since its initial description in 1967 and management is still largely supportive, with no established therapies targeted at the primary disease processes (Ashbaugh et al., 1967). Accordingly, there is a need for methods of early detection (Janz and Ware, 2013). There has been recent recognition of the clinical and biological heterogeneity within ARDS (Dowdy et al., 2006; Sweeney et al., 2018; Yehya et al., 2019) that reflects our incomplete understanding of the biology of ARDS.

Acute Respiratory Distress Syndrome (ARDS) is an illness that typically develops in people who are significantly ill or have serious injuries. Within a few hours, patients with ARDS will develop severe shortness of breath, low blood pressure, and unusually rapid breathing (Mayo Clinic, 2020). ARDS is characterized by fluid build-up that occurs in the alveoli of the lungs. The buildup of fluid prevents the lungs from filling up with air which results in less oxygen reaching the bloodstream (Katzenstein et al., 1976; Matute-Bello et al., 2008; Johns Hopkins Medicine, ). The lack of sufficient oxygen explains why patients with ARDS are placed on supplemental oxygen for milder symptoms while severe cases are placed in a mechanical ventilation system. ARDS is also a systemic inflammatory disease which suggests that while it is typically found to affect the respiratory system, it tends to affect other organ systems as well. The risk of death from ARDS increases with age and severity of illness while those that survive may experience lasting damage to their lungs (Ashbaugh et al., 1967; Force et al., 2012). The most common cause of ARDS is sepsis. Sepsis is characterized by a serious and widespread infection of the bloodstream. Another common cause of ARDS is severe pneumonia. A more recent cause of ARDS are patients that develop a severe case of COVID-19. These types of cases where patients develop ARDS can often be fatal, and those that do survive and recover from ARDS may have lasting pulmonary scarring (Ware and Matthay, 2000; Rubenfeld et al., 2005; Johns Hopkins Medicine, ).

Additional contributions to the knowledge about inheritance of ARDS and/or pathogenesis will be of great benefit in moving forward with successful clinical translation of new diagnostic, preventive, and therapeutic strategies (Vincent et al., 2006; Constantin et al., 2010; Tejera et al., 2012; Chiumello and Marino, 2017).

The NIH-NHLBI ARDS Network was a research network formed to study treatment of Acute Respiratory Distress Syndrome in 1994. The goal of the Network was to efficiently test promising agents, devices, or management strategies to improve the care of patients with ARDS. During its 20 years of service, 5,527 patients were enrolled in 10 randomized controlled trials and one observational study. Additional trials informed best practices by suggesting no role for routine use of corticosteroids, beta agonists, pulmonary artery catheterization, or early full calorie enteral nutrition. The ARDS Network also developed new outcome measures (ventilator free days) and promoted innovative and efficient techniques (factorial designs and coenrollment) to speed the discovery of new treatment approaches for patients with ARDS (ARDS Network, ). This network provided a robust amount of specimen for research experiments and has enabled the research community access to request these samples for secondary analysis.

NCBI GEO contains ∼222 ARDS patient samples from high throughput sequencing experiments, some of which utilize specimen derived as part of the ARDS network project. NCBI GEO serves as a resource to support the deposition of datasets from multiple sequencing platform options and accommodates a variety of sample groupings and associated metadata (National Center for Biotechnology Information, ). Another NCBI resource, dbGAP, as of the time for this report contained 2 published datasets from sequencing studies done in ARDS. Both GEO and dbGAP do not provide a direct output file containing primary level curated genes, gene functions, chromosomal tags, reference paper id and associated variants from published ARDS studies. The process of extraction of these types of gene lists from external resources and the data parsing required for secondary analysis and follow up computational work is often cumbersome and requires sophisticated Bioinformatics approaches. Here we present, ARDS DB, a comprehensive database for genes and variants specifically related to ARDS. The ARDS DB framework provides gene and variant information and associated metadata derived from primary level curation of experimentally verified studies. The caveat of a dedicated gene database for deeper analysis of ARDS is that it provides the user with a centralized location to retrieve pertinent information. ARDS DB is freely available via an open-source repository and represents a major step towards filling a gap in computational resources for bench biologists and clinicians.

## Materials and Methods

**FIGURE 1:**
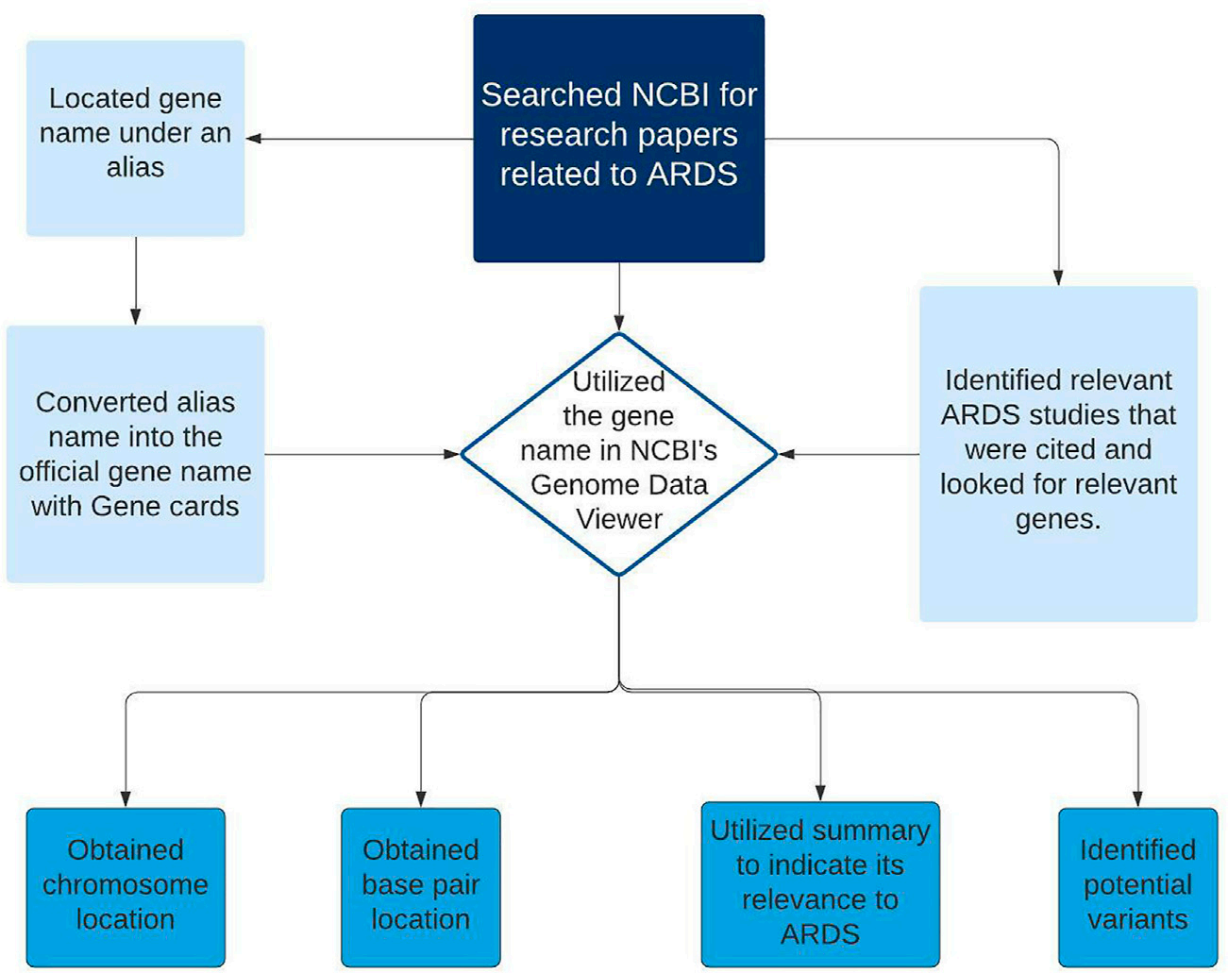
Diagram showing the workflow steps (data collection and primary curation) for the development of ARDS DB.

The data extraction process for the development of version 1 ARDS DB began in June of 2020 and consisted of 2 phases (Figure 1). The first phase consisted of retrieval of the related information obtained from 222 samples deposited at NCBI GEO with their associated papers published in Pubmed resource. Next, during the second phase a relevance text mining algorithm was employed via PubMed. The algorithm was based on the standard PubMed Best Match sort using a weighted term frequency algorithm. This approach calculates the frequency with which terms, in this case, Acute Respiratory Distress Syndrome, appear in PubMed records. Those frequencies are then applied in a weighted fashion to return a ranked list of PubMed citations that match the query terms.

An updated feature of the algorithm includes machine learning to re-rank the top articles returned. This algorithm combines over 150 signals that are helpful for finding best matching results. Most of these signals are computed from the number of matches between the search terms and the PubMed record, while others are either specific to a record (e.g., publication type; publication year) or specific to a search (e.g., search length). The new ranking model was built on relevance data obtained from anonymous PubMed search logs that were aggregated over an extended period of time (pubmed reference). The data was filtered by search term and species to include only those results pertaining to humans. Of the 202 articles identified, a manual curation process was employed to extract gene and/or variant lists. A comprehensive literature review was built into the process to compile gene lists. This search strategy was repeated across all matching articles. The relevant files for each gene sets or variants lists were extracted and further parsed using statistical analysis.

Statistical assessments were performed for each of these extracted lists using the R Bioconductor package (R Core Team, 2017). The criteria applied for the statistical evaluation was ≥1.5 fold change and *p* ≤ 0.05. Genes and variants found to be statistically significant were included in the final criteria. For each gene that was listed in the database, the corresponding PMID of the research article was included. www.genecards.org was used to obtain the official gene name as well as any other corresponding alias name for the gene (GeneCards,). Both the gene name from the published study and all other alias names for that gene were included in the database. Using the official gene name, the chromosome and position of the gene were also included as part of the metadata assembled. The gene names were then verified by direct import into “NCBI’s genome data viewer” and conducting a search for the gene. A summary of each gene’s function, as well as its chromosomal location and the start and stop site on the chromosome were documented. DAVID analysis was further employed to extract detailed gene description (DAVID, ). For analysis within DAVID, the “Entrez_gene_ID” option was selected as the identifier name. The gene lists submitted were converted into official gene symbol as part of the curation tasks.

The gene’s relation to ARDS patient outcomes is indicated in the database as provided in the published study. The two categories for patient outcomes included in ARDS DB are increased susceptibility or mortality which was found to be associated with the differentially expressed genes. Some genes were reported to cause both increased susceptibility and mortality, which are indicated within the ARDS DB. In addition, the gene function in ARDS, pathogenesis related information was included if pertinent to the primary research article. Permanent digital object identifiers (DOIs) for the original research articles are provided for each entry. The database was designed using a structured query language (SQL) architecture and is publicly available via the Zenodo open source ecosystem. https://zenodo.org/record/4033491#.YQN_cY5Khyw.

## Dataset

ARDS-DB contains a total of 238 genes that were found to be differentially expressed in ARDS patients. It contains the following types of metadata: official gene symbol, as well as any alias names that the gene could be associated with; NCBI gene ID, Chromosomal location, start and stop sites, variant id where relevant, as well as its corresponding location and type, which is listed in the database. The reference and primary publication where each gene was found are listed in the database, as well as a summary of the gene function. Lastly, the association between the gene and patient outcomes is provided where pertinent as well as a summary of the relatedness of the gene to ARDS patients. The corresponding reference containing this information is provided.

A major strength of ARDS DB is that it has been created through a pipeline consisting of intensive manual curation efforts, combined with machine learning algorithms. The synergy of these 2 approaches will ensure ease of continuous update as new data is deposited. Another strength is that the resource conveys specificity for ARDS and will help researchers looking for a centralized location to search for genes and variants. The detailed information on chromosomal location allows for ease of searching against any novel variants being assessed as comparison. The gene function information provided enables the user to learn quick facts about the gene and its role in signaling processes. The inclusion of patient outcomes provides clinicians quick reference information that will be informative to place the gene or variant in context for further consideration.

Currently the database is provided in a downloadable SQL format, which requires the user to download and compile it locally using a SQL-based interface. To address this, our future plan is to migrate ARDS DB into a stand-alone web-based resource. We would like to provide a web interface with easy access for bench biologists and clinicians that will offer advanced search features as well as data analysis and visualization all within the same ecosystem. With the availability of ARDS DB, users will be able to categorize and further understand the gene relationships involved in ARDS and the associated variants from published studies. The availability of variant locations will facilitate the direct comparison with novel variants or unique cases of familial ARDS such as that reported recently (Toby et al., 2020). An additional use for the database is to identify genes for training set to help build machine learning (ML) models to elucidate variations in ARDS patient outcomes. ML based assessments (such as Clustering algorithms, Random forest algorithms) and methods to include specialized sequence data such as from RNA seq and specialized sequencing technologies will be of particular focus. Potential associations of whole genome data to more specific patient cohorts for clinicians to better understand cases of familial ARDS will be of importance in future work.

## Dataset Description

The database is freely available in Zenodo and can be accessed through the following link: https://zenodo.org/record/4033491#.YPnI5BNKhQI or by searching within Zenodo for the following title: Acute Respiratory Distress Syndrome-Database of Genes (ARDS-DB). ARDS-DB is accessible via user download and can be viewed using a SQL-based interface such as MySQL (https://www.mysql.com/downloads/) or SQL-lite browser (https://sqlitebrowser.org/).

## Data Availability

The datasets presented in this study can be found in online repositories. The names of the repository/repositories and accession number(s) can be found in the article/Supplementary Material.

## References

[B1] ARDS Network. The NHLBI ARDS Network. Available at: http://www.ardsnet.org/ Accessed: July 1, 2020.

[B2] Ashbaugh D. Boyd Bigelow D. Petty T. Levine B. (1967). Acute Respiratory Distress in Adults. Lancet 290 (7511), 319–323. 10.1016/s0140-6736(67)90168-7 4143721

[B3] Chiumello D. Marino A. (2017). ARDS Onset Time and Prognosis: Is it a Turtle and Rabbit Race? J. Thorac. Dis. 9 (4), 973–975. 10.21037/jtd.2017.03.147 28523151 PMC5418263

[B4] Constantin J.-M. Grasso S. Chanques G. Aufort S. Futier E. Sebbane M. (2010). Lung Morphology Predicts Response to Recruitment Maneuver in Patients with Acute Respiratory Distress Syndrome. Crit. Care Med. 38 (4), 1108–1117. 10.1097/ccm.0b013e3181d451ec 20154600

[B5] DAVID. Functional Annotation Tools. Available at: david.ncifcrf.gov/tools.jsp

[B6] Dowdy D. W. Eid M. P. Dennison C. R. Mendez-Tellez P. A. Herridge M. S. Guallar E. (2006). Quality of Life after Acute Respiratory Distress Syndrome: a Meta-Analysis. Intensive Care Med. 32 (8), 1115–1124. 10.1007/s00134-006-0217-3 16783553

[B7] Force A. D. T. Ranieri V. M. Rubenfeld G. D. Thompson B. T. Ferguson N. D. Caldwell E. (2012). Acute Respiratory Distress Syndrome: the Berlin Definition. JAMA 307 (23), 2526–2533. 10.1001/jama.2012.5669 22797452

[B8] GeneCards. GeneCards®: The Human Gene Database. Available at: www.genecards.org/ Accessed: July 1, 2020.

[B9] Janz D. R. Ware L. B. (2013). The Needle in the Haystack: Searching for Biomarkers in Acute Respiratory Distress Syndrome. Crit. Care 17 (5), 192. 10.1186/cc13025 24073648 PMC4056564

[B10] Johns Hopkins Medicine. COVID-19 Lung Damage. Available at: www.hopkinsmedicine.org/health/conditions-and-diseases/coronavirus/what-coronavirus-does-to-the-lungs .

[B11] Katzenstein A. L. Bloor C. M. Leibow A. A. (1976). Diffuse Alveolar Damage-Tthe Role of Oxygen, Shock, and Related Factors. A Review. Am. J. Pathol. 85 (1), 209–228. 788524 PMC2032554

[B12] Matute-Bello G. Frevert C. W. Martin T. R. (2008). Animal Models of Acute Lung Injury. Am. J. Physiol. Lung Cell Mol. Physiol. 295 (3), L379–L399. 10.1152/ajplung.00010.2008 18621912 PMC2536793

[B13] Mayo Clinic (2020). ARDS. Mayo Foundation for Medical Education and Research. Available at: www.mayoclinic.org/diseases-conditions/ards/symptoms-causes/syc-20355576 Accessed: June 13, 2020.

[B14] National Center for Biotechnology Information. National Center for Biotechnology Information. U.S. National Library of Medicine. Available at: www.ncbi.nlm.nih.gov/ .

[B15] Phua J. Badia J. R. Adhikari N. K. J. Friedrich J. O. Fowler R. A. Singh J. M. (2009). Has Mortality from Acute Respiratory Distress Syndrome Decreased over Time? Am. J. Respir. Crit. Care Med. 179 (3), 220–227. 10.1164/rccm.200805-722oc 19011152

[B16] R Core Team (2017). R: A Language and Environment for Statistical Computing. Vienna, Austria: R Foundation for Statistical Computing. Available at: https://www.R-project.org/ .

[B17] Rubenfeld G. D. Caldwell E. Peabody E. Weaver J. Martin D. P. Neff M. (2005). Incidence and Outcomes of Acute Lung Injury. N. Engl. J. Med. 353 (16), 1685–1693. 10.1056/nejmoa050333 16236739

[B18] Sweeney T. E. Thomas N. J. Howrylak J. A. Wong H. R. Rogers A. J. Khatri P. (2018). Multicohort Analysis of Whole-Blood Gene Expression Data Does Not Form a Robust Diagnostic for Acute Respiratory Distress Syndrome. Crit. Care Med. 46 (2), 244–251. 10.1097/ccm.0000000000002839 29337789 PMC5774019

[B19] Tejera P. Meyer N. J. Chen F. Feng R. Zhao Y. O'Mahony D. S. (2012). Distinct and Replicable Genetic Risk Factors for Acute Respiratory Distress Syndrome of Pulmonary or Extrapulmonary Origin. J. Med. Genet. 49 (11), 671–680. 10.1136/jmedgenet-2012-100972 23048207 PMC3654537

[B20] Toby I. T. Thomas N. J. Thorenoor N. Spear D. DiAngelo S. Floros J. (2020). Characterizing a Focused Landscape of Familial Acute Respiratory Distress Syndrome. Biomark Applic. 04, 141. 10.29011/2576-9588.100041

[B21] Vincent J.-L. Sakr Y. Sprung C. L. Ranieri V. M. Reinhart K. Gerlach H. (2006). Sepsis in European Intensive Care Units: Results of the SOAP Study. Crit. Care Med. 34 (2), 344–353. 10.1097/01.ccm.0000194725.48928.3a 16424713

[B22] Ware L. B. Matthay M. A. (2000). The Acute Respiratory Distress Syndrome. N. Engl. J. Med. 342 (18), 1334–1349. 10.1056/nejm200005043421806 10793167

[B23] Wellman T. J. de Prost N. Tucci M. Winkler T. Baron R. M. Filipczak P. (2016). Lung Metabolic Activation as an Early Biomarker of Acute Respiratory Distress Syndrome and Local Gene Expression Heterogeneity. Anesthesiology 125 (5), 992–1004. 10.1097/aln.0000000000001334 27611185 PMC5096592

[B24] Yehya N. Thomas N. J. Wong H. R. (2019). Evidence of Endotypes in Pediatric Acute Hypoxemic Respiratory Failure Caused by Sepsis. Pediatr. Crit. Care Med. 20 (2), 110–112. 10.1097/pcc.0000000000001808 30720645 PMC6996087

